# Intravitreal faricimab in patients with aflibercept-refractory neovascular age-related macular degeneration: short and long-term outcomes and assessment of volume dynamics using an artificial intelligence-based tool

**DOI:** 10.1186/s40942-025-00751-9

**Published:** 2025-11-19

**Authors:** Mickael Barbosa, Nicolò Bartolomeo, Yannic Pannatier Schuetz, Anna Chiara Nascimbeni, Daniela Gallo Castro, Mamadou Pathé Barry, Aude Ambresin

**Affiliations:** Swiss Visio Retina Research Center, Swiss Visio Montchoisi, Avenue du Servan 38, Lausanne, 1006 Switzerland

**Keywords:** Age-related macular degeneration, Neovascular, Switch, Faricimab, Outcomes, Artificial intelligence

## Abstract

**Purpose:**

This study assessed the short- and long-term outcomes of intravitreal (IVT) faricimab treatment in patients with neovascular age-related macular degeneration (nAMD) refractory to aflibercept. The main aim was to investigate whether faricimab might enable longer treatment intervals versus aflibercept through improved fluid control, evaluated through use of an artificial intelligence-based quantification tool to evaluate retinal fluid dynamics.

**Methods:**

This observational cohort study involved patients with refractory nAMD who received at least three consecutive IVT aflibercept 2.0 mg injections before switching to IVT faricimab (with a four-month loading phase followed by a treat-and-extend regimen) due to persistent or recurrent disease despite 4–8-week treatment intervals. Functional and anatomical outcome measures were recorded, and fluid volume dynamics were quantified, at baseline, monthly to Month 4, and at Months 6, 9, and 12.

**Results:**

Seventy-four eyes from 60 patients were included, with a mean ± standard deviation duration of prior aflibercept therapy of 24 ± 17 months. Fifty-two eyes completed 12-month follow-up. At Month 12, mean best-corrected visual acuity showed no significant change from baseline (+ 0.01 Early Treatment of Diabetic Retinopathy Study letters, *p* = 0.64). Significant reductions in mean central retinal thickness (− 80.8 μm, *p* = 0.0001) and maximal pigment epithelium detachment (PED) height (− 28.2 μm, *p* = 0.011), were observed at Month 4 and maintained to Month 12. Mean fluid volumes (intraretinal fluid [IRF], subretinal fluid [SRF]), and PED decreased significantly at Month 4 (− 26.3 nL, *p* = 0.007; −41.5 nL, *p* = 0.0001; and − 175.4 nL, *p* = 0.0001, respectively). At Month 12, reductions in IRF and PED volumes were sustained. The maximal fluid-free interval increased from 4.4 weeks, prior to switching to faricimab, to 6.5 weeks (*p* = 0.001) after switching, while mean last treatment interval improved from 5.0 ± 1.4 weeks at baseline to 7.3 ± 2.6 weeks at month 12 (*p* < 0.0001).

**Conclusion:**

Faricimab may offer a valuable alternative for patients with refractory nAMD. The use of four loading injections administered monthly, followed by a treat-and-extend regimen can result in maintenance of visual acuity and improve anatomical parameters and retinal fluid activity, allowing for longer treatment intervals.

**Supplementary Information:**

The online version contains supplementary material available at 10.1186/s40942-025-00751-9.

## Key messages

**What is known:**

The current standard of care for neovascular age-related macular degeneration (nAMD) involves intravitreal injection of vascular endothelial growth factor inhibitors, which have proven effective. However, substantial unmet needs remain, particularly for improved efficacy and longer duration of action, especially in patients with refractory disease.

**What is new:**

In patients with refractory nAMD, mean best-corrected visual acuity showed no significant change from baseline following switch from aflibercept 2.0 mg to faricimab. However, there were reductions from baseline to Month 12 in mean central retinal thickness and maximal pigment epithelium detachment (PED) height. A novel artificial intelligence-based automated quantification tool was used to assess fluid volume dynamics and determined a decrease in mean IRF, SRF and PED volumes from baseline to Month 4.

The most significant anatomical effect of faricimab on IRF and SRF volumes was achieved after the first injection. However, a continuous decrease in PED volume was observed during the loading phase, suggesting that the four monthly loading dose injections may be necessary for patients with refractory nAMD.

**Supplementary Information:**

The online version contains supplementary material available at 10.1186/s40942-025-00751-9.

## Introduction

Age-related macular degeneration (AMD) is a leading cause of vision impairment and blindness worldwide in patients aged >60 years. The neovascular form of AMD (nAMD) is characterized by growth of new blood vessels called macular neovascularization (MNV), which can lead to hemorrhage and retinal fluid accumulation [1].

The current standard of care for nAMD is intravitreal (IVT) injection of vascular endothelial growth factor (VEGF) inhibitors [2], including traditional agents such as ranibizumab (Lucentis^®^, Novartis), brolucizumab (Beovu^®^, Novartis), and aflibercept 2.0 mg (Eylea^®^; Bayer). Landmark clinical trials have demonstrated the efficacy of these treatments in patients with nAMD [3–6]. However, despite their success, several unmet needs persist, including the need for improved efficacy, longer duration of action (reducing injection frequency), improved effectiveness on angiogenesis and vascular permeability, and reduced scarring [2]. Recently, newer nAMD treatments such as faricimab and high-dose aflibercept (8 mg) have been introduced to reduce treatment burden [7–9].

Faricimab (Vabysmo^®^, Roche/Genentech) is the first bispecific monoclonal antibody designed for IVT use that uniquely inhibits two signaling pathway components involved in nAMD: VEGF and angiopoietin-2 (Ang-2) [7, 10]. It has been approved for the treatment of nAMD and diabetic macular edema since January 2022 [11, 12]. Angiopoietin-2 is a novel therapeutic target that has a role in inflammation and vascular stability, specifically mediated through a transmembrane receptor, Tie2, located on endothelial cells [10, 13–15].

The pivotal phase 3 clinical trials TENAYA (NCT03823287) and LUCERNE (NCT03823300) were parallel, multicenter, double-blind studies designed to evaluate the efficacy and safety of faricimab in treating nAMD. Faricimab, administered with a treatment interval of up to 16 weeks following a loading phase of four monthly IVT injections, demonstrated non-inferiority to aflibercept in terms of mean change in best-corrected visual acuity (BCVA) and central retinal thickness (CRT) from baseline to Weeks 40, 44, and 48 [7]. Rates of ocular adverse events were also similar between faricimab and aflibercept [7]. Several real-world studies have demonstrated the clinical benefit of initiating faricimab therapy in treatment-naïve [16–18] and pre-treated patients [18–27]. Therefore, switching to IVT faricimab in patients with refractory nAMD previously treated with aflibercept may improve functional and anatomical responses to treatment.

The aim of this study was to evaluate the early and 12-month outcomes of IVT faricimab treatment, including treatment intervals, and to assess the fluid volume dynamics using an artificial intelligence-based automated quantification tool, in patients with refractory nAMD previously treated with aflibercept. We were particularly interested in determining whether faricimab facilitates longer treatment intervals due to enhanced fluid control.

## Methods

This was an observational, retrospective, single-arm, single-center cohort study. The study was approved by the local ethics committee (CER-VD; BASEC 2023 − 00814). Patients attending the Swiss Visio Montchoisi Ophthalmology Center in Lausanne, Switzerland, from September 2022 to July 2023, and who met the following inclusion criteria were enrolled in the study: (1) previously treated with at least three consecutive IVT aflibercept 2.0 mg injections and switched to IVT faricimab injections, (2) a mean dosing interval between the last IVT aflibercept dose and the first IVT faricimab dose of < 60 days, (3) a baseline BCVA on the Early Treatment of Diabetic Retinopathy Study (ETDRS) chart of ≥ 25 and ≤ 85 letters, and (4) had switched from aflibercept to faricimab due to either refractive disease activity on spectral domain optical coherence tomography (SD-OCT) despite treatment with aflibercept at 4–6-week treatment intervals or recurrent disease activity despite treatment with aflibercept at 6–8-week treatment intervals. The termination date was defined as the date the last included patient completed their 12-month follow-up visit. Disease activity was defined as the new or persistent presence of any amount of retinal fluid, including intraretinal fluid (IRF), subretinal fluid (SRF), or sub–retinal pigment epithelium (sub-RPE) fluid. Pigment epithelium detachment (PED) was also considered a marker of activity if an increase in its height or width was observed prior to the switch. Its mere presence was not considered a sign of active disease. Retinal hemorrhage and subretinal hyperreflective material (SHRM) were also regarded as indicators of activity. Exclusion criteria included the presence of neovascularization caused by any other pathologies, if the switch to faricimab was due to an adverse event or intolerance to aflibercept injections, laser treatments performed at any time or photodynamic therapy up to 3 months before the switch, and if there was any concomitant ocular disease that could interfere with the therapeutic response, visual acuity, or interpretation of the study results. Previous use of any other VEGF inhibitor was not an exclusion criterion if at least the last three injections before the switch were with aflibercept. Both eyes could be included in the study if the eligibility criteria were fulfilled. Types 1, 2 and 3 macular neovascularization MNV were included in the study, as well as polypoidal choroidal vasculopathy (PCV). MNV type was evaluated using multimodal imaging including OCT, OCT-angiography and previous fluorescein/indocyanine green angiography, based on the classification proposed by Spaide and al [28]. Type 1 MNV was defined as RPE elevation with heterogeneous material, type 2 MNV as neovascular complex above the RPE, in the subretinal space, and type 3 MNV as an extension of hyperreflectivity into the outer retina. PCV was diagnosed based on previous indocyanine green angiography (ICGA) findings, which is routinely performed at the time of initial diagnosis in our specialized center.

Patients followed a treatment regimen comprising a loading phase of four monthly injections of faricimab followed by a treat-and-extend regimen in which treatment intervals could be extended by 2 weeks at the investigators’ discretion, based on the presence or absence of IRF, SRF, and sub-RPE fluid/PED activity, as well as BCVA assessments, to a maximum treatment interval of 16 weeks. The treat-and-extend regimen permitted an extension in treatment interval by 2 weeks if there was no IRF, SRF, or sub-RPE fluid and no increase in maximal PED height; the interval was reduced by 2 weeks if there was new or an increase in volume of IRF, SRF, or sub-RPE fluid. No additional photodynamic therapy (PDT) was performed in patients with PCV during the follow-up period.

Assessments were undertaken from the electronic medical record of our center at baseline (before switching therapy from aflibercept to faricimab), monthly up to Month 4 (final loading dose), and at Months 6, 9, and 12. BCVA was measured using decimal Snellen chart, as per routine clinical practice, and subsequently converted into ETDRS letters. Outcome measures assessed included BCVA, CRT, maximal PED height, presence of IRF, SRF, and sub-RPE fluid on SD-OCT, using the Heidelberg SPECTRALIS^®^ system (Heidelberg Engineering, Heidelberg, Germany). Maximal PED height was defined as the greatest vertical separation between the elevated RPE and Bruch’s membrane, measured where PED appeared tallest at baseline. Scans were centered on the fovea using a 6 × 6 mm macular cube consisting of 97 B-scans, each consisting of 10 ART frames to ensure a high imaging resolution. All B-scans were systematically reviewed at each visit for every eye to assess the presence of any retinal fluid. This manual scrolling procedure was applied consistently for all eyes and across all study visits. Baseline anatomical characteristics such as MNV type, PED type, outer retinal tubulation, subretinal hyperreflective material, hyperreflective foci and RPE tear, were also identified from OCT images. PEDs were classified into four types based on their internal reflectivity patterns on OCT. Serous PED was defined as a homogeneous, hyporeflective elevation of the RPE with a smooth contour and without presence of internal reflectivity. Fibrovascular PEDs consisted entirely of hyperreflective fibrovascular tissue without any serous component. Mixed PEDs contained both serous fluid and fibrovascular tissue, with predominantly serous PED consisting of ≥ 50% serous fluid, and fibrovascular predominant PEDs consisting of ≥ 50% fibrovascular tissue. In contrast, sub-RPE fluid was defined as any fluid accumulation in that space below the RPE.

For the volumetric analysis we used the validated and CE-marked artificial intelligence-based RetinAI Discovery platform (Ikerian AG, Switzerland, license version 4.6). All OCT cube images were transferred to a secure cloud-based system and then processed by the software for automated quantification of IRF, SRF and PED volumes. Data on performance and repeatability of fluid detection, has been previously published [29, 30]. Moreover, real-world studies have reported the outcomes of anti-VEGF therapy using this tool [31, 32]. All values were used as provided by the software, and no segmentation adjustments were performed.

Baseline and demographic characteristics are summarized using descriptive statistics. Data from eyes who did not terminate the follow up period were excluded from the analysis after treatment discontinuation. Non-parametric Wilcoxon signed-rank tests were used to compare changes between continuous variables, and Chi-squared tests were used to compare changes between qualitative variables. All statistical analyses were performed using Stata (version 12.0). Statistical significance was defined as a *p* value < 0.05. Results are shown as the mean ± standard deviation (SD) for continuous variables and proportions for categorical variables.

## Results

This study included 74 eyes from 60 patients with a mean (± SD) age of 80 (± 8) years. Most patients were female (40/61; 66.7%). Patients received aflibercept 2.0 mg therapy for a mean of 24 (± 17) months, with a mean dosing interval between the last IVT aflibercept dose and the first IVT faricimab dose of 5.0 (± 1.4) weeks, and a mean dosing interval between the last three IVT aflibercept doses and the first IVT faricimab dose of 5.0 (± 1.1) weeks. Before switching to faricimab, patients had received a mean 21 (± 14) consecutive aflibercept 2.0 mg injections over a mean duration of 24 (± 17) months (Supplementary Table 1). The majority of patients had type 1 MNV (44/74, 59.5%) and PED type mixed, predominantly fibrovascular (Supplementary Table 2), Additional baseline and anatomical characteristics before switching to faricimab are provided in Supplementary Tables 1 and 2, respectively. Fifty-two eyes completed the 12-month follow-up. All eyes completed the loading phase, while 6 eyes were excluded afterward because of deviations from the predefined treatment protocol.

Supplementary Fig. 1 shows the changes in mean BCVA, CRT, and maximal PED height from baseline to Month 12. At Month 12, mean BCVA showed no significant change from baseline (+ 0.01 ETDRS letters, *p* = 0.64). For patients with 12-month data, the reductions versus baseline in mean CRT (− 80.8 μm, *p* = 0.0001) and mean maximal PED height (− 28.2 μm, *p* = 0.011) were maintained to Month 12.

**Fig. 1:**
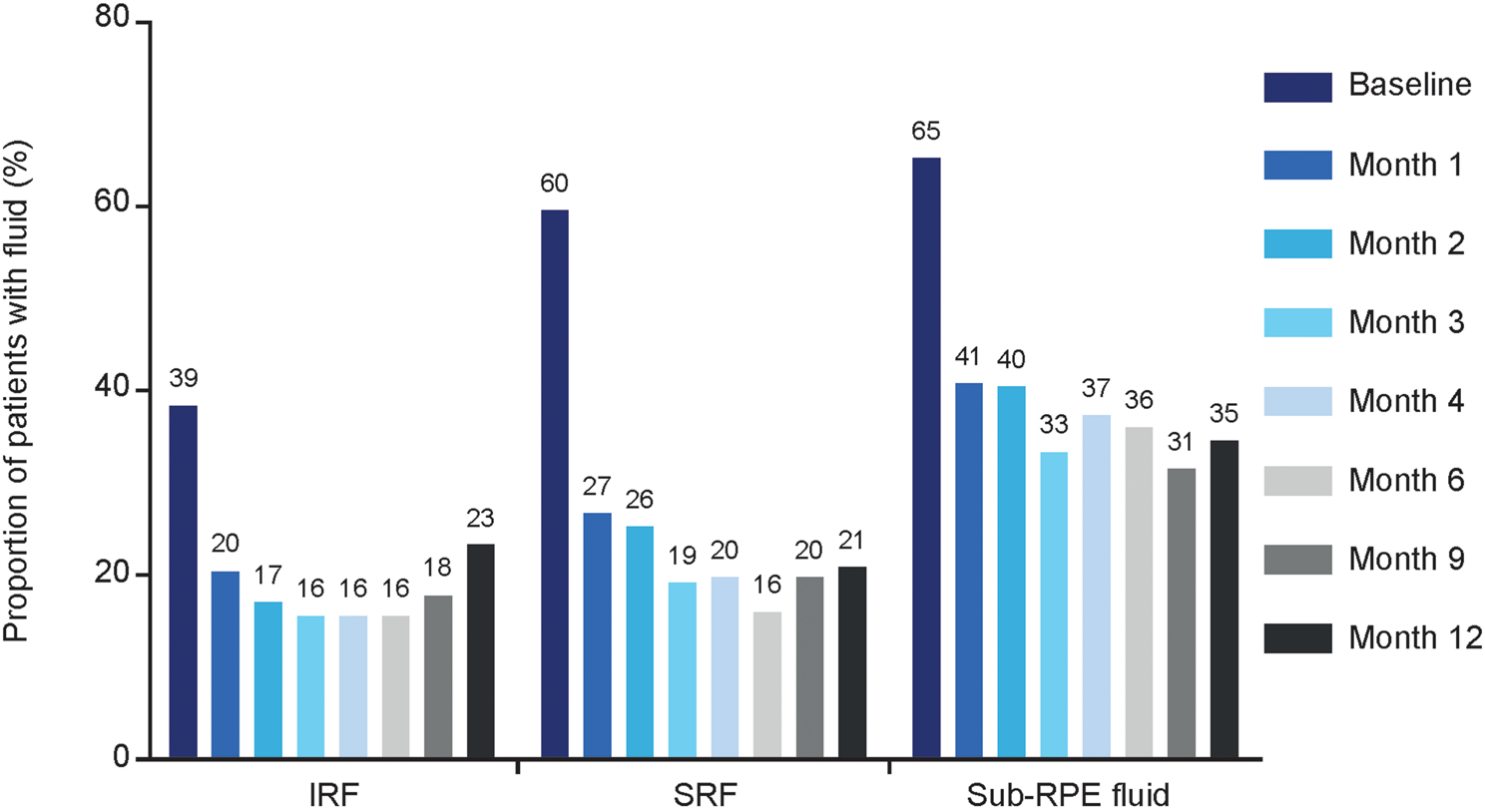
Proportion of patients with fluid (IRF, SRF, and sub-RPE fluid) from baseline to Month 12. *IRF* intraretinal fluid, *RPE* retinal pigment epithelium, *SRF* subretinal fluid

**Fig. 2:**
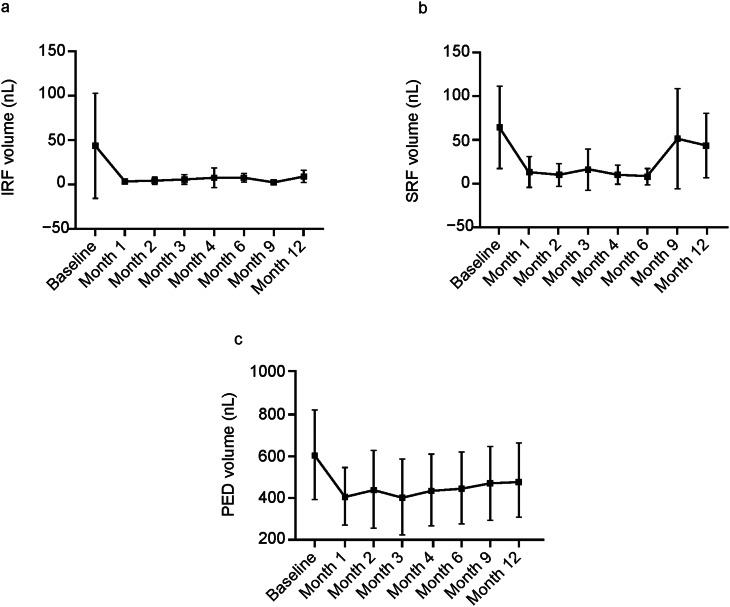
Dynamics of retinal fluid volumes from baseline to Month 12: change in mean ± SD (**a**) IRF, (**b**) SRF, and (**c**) PED volumes. *IRF* intraretinal fluid, *PED* pigment epithelium detachment, *SRF* subretinal fluid

In patients with 12-month data, IRF was present in 38.5% of eyes, SRF in 59.6% of eyes, and sub-RPE fluid in 65.4% of eyes at baseline, before switching to faricimab; all these proportions were significantly decreased at Month 4 after switching, with fluid present in 15.7%, 19.6%, and 37.3% of eyes, respectively. At Month 12, the proportion of eyes with IRF was 23.1%, with SRF was 21.2%, and with sub-RPE fluid was 34.6% (Fig. 1). Mean IRF, SRF, and PED volumes decreased by − 26.3 nL (*p* = 0.007), − 41.5 nL (*p* < 0.001), and − 175.4 nL (*p* < 0.001) from baseline to Month 4, respectively (Supplementary Fig. 2); for the 12-month cohort, decreases in IRF and PED volume were sustained (Fig. 2). An increase in SRF volume was observed from Month 9 due to some patients experiencing fluid recurrence while the treatment interval was extended (Fig. 2).

**Fig. 3:**
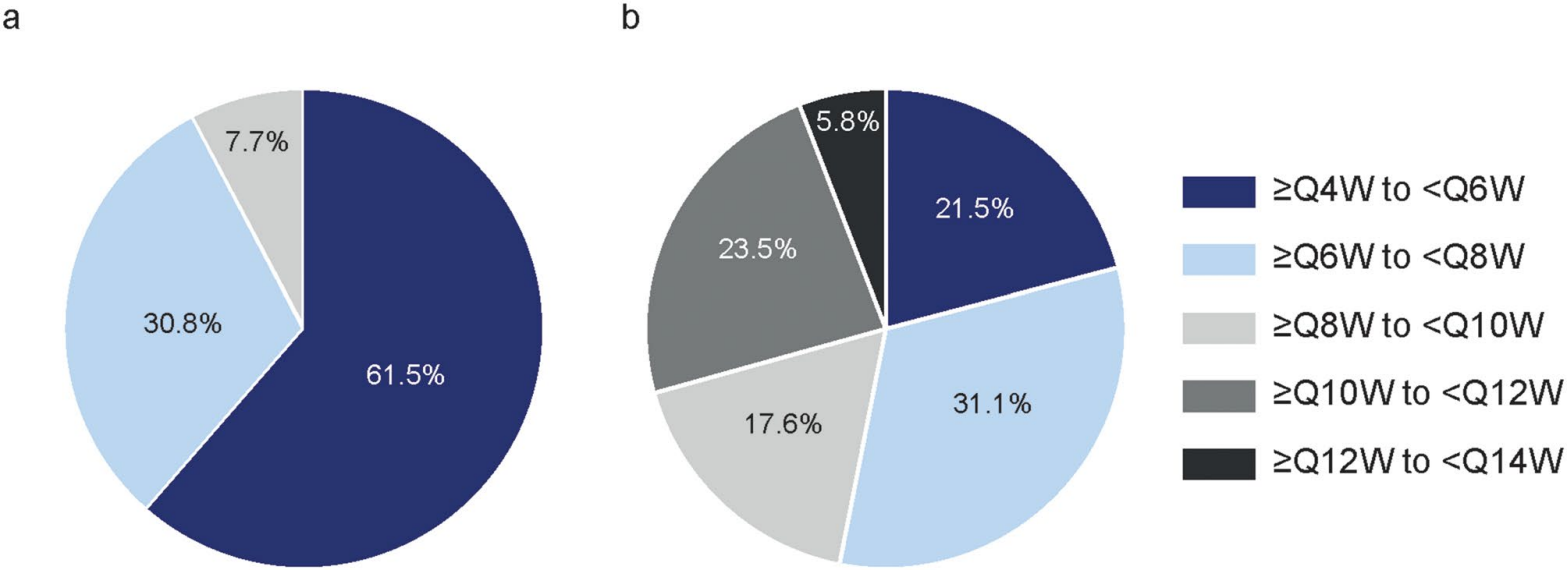
Proportion of patients within each treatment interval (**a**) before (baseline) and (**b**) after treatment switch (Month 12). *QXW* every X weeks

Before switching to faricimab, most patients (61.5%) were receiving aflibercept with a treatment interval of 4–6 weeks. However, by Month 12 of this study, only 21.5% of patients continued on this treatment interval with faricimab and almost half of patients (46.9%) had a treatment interval of > 8 weeks (Fig. 3). The mean maximal fluid-free interval with aflibercept (defined as the longest treatment interval with fluid-free OCT in the last 6 months before switching) was 4.4 (± 0.9) weeks and the maximal fluid-free interval during the 12-month follow-up with faricimab increased to 6.5 (± 2.4) weeks (*p* = 0.001). The mean last treatment interval before switching to faricimab was 5.0 (± 1.4) weeks, and it increased to 7.3 (± 2.6) weeks (*p* < 0.0001) at month 12. Supplementary Fig. 3 illustrates the distribution of patients by treatment interval at baseline and at 12 months after initiating faricimab.

Treatment was discontinued in 22 eyes (29.7%) during the observational period. Reasons of discontinuation were: occurrence of adverse events (3 eyes, 4.1%), loss to follow up (6 eyes, 8.1%), switch to another therapy (3 eyes, 4.1%), deviation from protocol (5 eyes), treatment cessation following a stroke in two patients (3 eyes, 4.1%) and death from one patient (2 eyes, 2.7%).

A mild sterile vitritis developed in one eye within 2 months of treatment and was successfully managed with topical therapy with no visual loss. One patient experienced a vitreous hemorrhage after the second injection, leading to the suspension of treatment in both eyes. No significant ocular events (e.g. endophthalmitis, retinal detachment) were reported in any of the eyes studied.

## Discussion

In this real-world study, we demonstrated that switching from aflibercept 2.0 mg to faricimab resulted in maintenance of visual acuity and significant improvement in anatomical parameters and retinal fluid activity, both in the short and long-term, in patients with persistent or recurrent nAMD. We also demonstrated the use of a novel validated artificial intelligence-based automated quantification tool to evaluate fluid volume dynamics, adding to the literature supporting the practical application of artificial intelligence to rapidly and objectively quantify retinal fluid [33, 34]. Retinal fluid volumes are used as a proxy for response to treatment in eyes with nAMD, and AI-based tools provide rapid and objective measurements, reducing manual effort and human error while allowing personalization of treatment [35].

In our study, after a loading phase of four monthly faricimab injections, there was a significant reduction in retinal fluid compartment volumes (IRF and SRF) and PED volume from baseline. At Month 12, mean IRF, SRF, and PED volumes remained significantly decreased from baseline. In addition, reductions were observed in the mean CRT and maximal PED height after the loading dose and at Month 12, and there was a low proportion of eyes with SRF at Month 12 (11/52, 21.2%).

Our results are consistent with the first real-world studies evaluating faricimab efficacy in previously treated patients with nAMD. The TRUCKEE study showed a significant reduction in the proportion of eyes with IRF or SRF, as well as an improvement in mean CRT and maximal PED height in both treatment-naïve and pre-treated patients with nAMD who switched from aflibercept [18]. Similarly, a recent study showed a reduction in the proportion of eyes with SRF and IRF in patients who switched to faricimab [19], and another study found a significant improvement in mean CRT and maximal PED height after three faricimab injections in patients with nAMD who switched from aflibercept [20].

Most of the improvements in the measured anatomical outcomes of this study were achieved at Month 1 after the first loading dose of faricimab, with no difference in IRF and SRF volumes between the first and second faricimab injections. In line with these results, a recent retrospective study that compared two subgroups of patients with nAMD who switched to IVT faricimab every 8 weeks or IVT faricimab every 12 weeks also found no significant difference in IRF and SRF volumes [27]. Similarly, a *post hoc* analysis of the head-to-head dosing phase of TENAYA and LUCERNE showed that retinal dryness, as measured by the absence of SRF and IRF was observed in 75% patients, and was achieved by Week 8 with faricimab versus Week 12 with aflibercept [36]. These data suggest that the drying effect is greater and occurs faster with faricimab versus aflibercept. In our study, a continuous decrease in PED volume was observed during the loading phase, suggesting that a full loading dose phase of faricimab, comprising of four monthly injections, may be necessary in patients with refractory nAMD to optimize fluid resolution. However, the relevance of PED for visual function is controversial and fluid and PED resolution has not been found to correlate with improved visual function [37].

Despite the anatomical improvements in our study, these changes were not associated with improved visual acuity scores. This could be explained by the chronic nature of the disease, as evidenced by the high mean number of previous injections before switching therapy, which could have led to subsequent damage to the RPE and photoreceptors. Additionally, the presence of outer retinal tubulation, subretinal hyperreflective material, and RPE tears at baseline may indicate a more advanced disease stage. Lastly, although faricimab acts through two pathways (targeting both Ang-2 and VEGF), any significant change in visual acuity was not likely because of a possible ceiling effect, given the given the already relatively good mean baseline BCVA score in this patient cohort. Moreover, it remains unclear whether continuing aflibercept therapy instead of switching would have resulted in poorer visual outcomes over time. Although the anatomical outcomes appear satisfactory, the lack of visual improvement raises concerns regarding the clinical relevance of such results. Other parameters of subjective or objective function, such as low-luminance visual acuity, contrast sensitivity, reading speed and microperimetry should also be considered as visual parameters. Incorporating such visual function tests in comparative studies of switch and non-switch cohorts may potentially reveal different responses in visual outcomes.

Most patients were able to extend their treatment intervals due to the effectiveness of faricimab at reducing fluid volumes, with approximately 50% transitioning to intervals of > 8 weeks, demonstrating the potential for prolonged treatment intervals with faricimab while maintaining efficacy.

There are several limitations to this study, including the single-center setting, small sample size – limiting further subgroup analyses – and lack of a control group. OCT analysis was performed by a single grader, potentially leading to subjective variability. The majority of study eyes had nAMD characterized by type 1 MNV, which may limit generalizability of the results of anti-VEGF therapy. Additionally, our study did not perform subgroup analyses according to early versus late switching of anti-VEGF therapy. Many studies have suggested that timing of switching affects treatment outcomes, but no consensus exists on the optimal strategy [38].

While aflibercept 2 mg has long been a standard comparator and was still largely used during the study period, aflibercept 8 mg has been introduced more recently. Therefore, a direct comparison between faricimab and aflibercept 8 mg should be considered in future studies. This study demonstrates the successful application of a novel artificial intelligence-based automated quantification tool to analyze fluid volume dynamics during the loading phase of faricimab treatment.

## Conclusion

Faricimab may be a valuable alternative when considering switching therapy in patients with refractory nAMD. A loading dose of faricimab followed by a treat-and-extend regimen resulted in maintenance of visual acuity alongside significant improvements in anatomical parameters and retinal fluid activity, while also allowing for extended treatment intervals. Longer follow-up is needed to determine whether these results can be maintained over time and to observe the treatment durability outcomes.

## Supplementary Information

Below is the link to the electronic supplementary material.

Supplementary Material 1

Supplementary Material 2

## Data Availability

No datasets were generated or analysed during the current study.
