# Supplementary material for: Intravitreal faricimab in patients with aflibercept-refractory neovascular age-related macular degeneration: short and long-term outcomes and assessment of volume dynamics using an artificial intelligence-based tool
Source: Int J Retina Vitreous. 2025 Nov 19;11:126. doi: 10.1186/s40942-025-00751-9 (PMC12628855; doi:10.1186/s40942-025-00751-9)
Supplement: Supplementary file 1 — Supplementary Material 1 [file 40942_2025_751_MOESM1_ESM.docx]

Supplementary Information contains baseline and anatomical characteristics prior to switching to faricimab, predictive factors associated with fluid volume changes following loading dose of faricimab, change in mean visual and anatomical characteristics from baseline to Month 12, fluid volume dynamics from baseline to Month 4, and maximal fluid-free treatment intervals before and after treatment switch.
