# Supplementary material for: Intravitreal faricimab in patients with aflibercept-refractory neovascular age-related macular degeneration: short and long-term outcomes and assessment of volume dynamics using an artificial intelligence-based tool
Source: Int J Retina Vitreous. 2025 Nov 19;11:126. doi: 10.1186/s40942-025-00751-9 (PMC12628855; doi:10.1186/s40942-025-00751-9)
Supplement: Supplementary file 2 — Supplementary Material 2 [file 40942_2025_751_MOESM2_ESM.docx]

**Supplementary Information**

**Intravitreal faricimab in patients with aflibercept-refractory neovascular age-related macular degeneration: short and long-term outcomes and assessment of volume dynamics using an artificial intelligence-based tool**

Mickael Barbosa, Nicolò Bartolomeo, Yannic Pannatier Schuetz, Anna Chiara Nascimbeni, Daniela Gallo Castro, Pathé Mamadou Barry, Aude Ambresin

Swiss Visio Retina Research Center, Swiss Visio Montchoisi, Lausanne, Switzerland

Corresponding author contact details: Aude Ambresin; Swiss Visio Retina Research Center, Swiss Visio Montchoisi, Avenue du Servan 38, 1006 Lausanne, Switzerland; tel: +41 58 274 2200; email: [aambresinsvrrc@swissvisio.net](mailto:aambresinsvrrc@swissvisio.net)

**Supplementary Table 1** Baseline characteristics before switching to faricimab (n=60 patients, 74 eyes)

|  |  |
| --- | --- |
| Sex, *n* (%)  Female  Male | 40 (66.7)  20 (33.3) |
| Mean ± SD age, years | 79.7 ± 7.6 |
| Mean ± SD number of previous IVT injections | 28 ± 17.0 |
| Mean ± SD number of consecutive IVT aflibercept 2.0 mg doses before switch | 21 ± 14.0 |
| Mean ± SD duration of aflibercept 2.0 mg therapy, months | 24 ± 17.0 |
| Mean ± SD dosing interval between last IVT aflibercept dose and first IVT faricimab dose, weeks | 5.0 ± 1.4 |
| Mean ± SD dosing interval between the last three IVT aflibercept doses and the first IVT faricimab dose, weeks | 5.0 ± 1.1 |

*IVT* intravitreal, *SD* standard deviation

**Supplementary Table 2** Anatomical characteristics before switching to faricimab (n=60 patients, 74 eyes)

|  | Patients, *n* (%) |
| --- | --- |
| Phakic status  Phakic  Pseudophakic | 58 (78.4)  16 (21.6) |
| MNV subtype  Type 1  Type 2  Type 3  PCV | 44 (59.5)  4 (5.4)  9 (12.2)  17 (23.0) |
| PED type  Absent  Drusenoid  Fibrovascular  Mixed PED predominantly fibrovascular  Mixed PED predominantly serous | 5 (6.7)  1 (1.3)  18 (24.3)  38 (51.3)  12 (16.2) |
| Outer retinal tubulations | 10 (13.5) |
| Subretinal hyperreflective material/fibrosis | 12 (16.2) |
| Hyperreflective foci | 33 (44.6) |
| RPE tear | 3 (4.0) |

*MNV* macular neovascularization, *PCV* polypoidal choroidal vasculopathy, *PED* pigment epithelium detachment, *RPE* retinal pigment epithelium

**Supplementary Table 3** Predictive factors associated with IRF, SRF, and PED volume changes following the loading dose

|  | Univariate | | Multivariate | |  |
| --- | --- | --- | --- | --- | --- |
|  | B coefficient (*p* value) | | B coefficient (*p* value) | |  |
| **Predictive factors associated with IRF volume change** | | | | |  |
| Age | −2.19 (0.505) | |  | |  |
| Sex | 37.66 (0.442) | |  | |  |
| Duration of aflibercept therapy | 1.48 (0.280) | |  | |  |
| MNV type |  | |  | |  |
| Type 1 | Reference | | Reference | |  |
| Type 3 | −236.5 (<0.001*) | | −9.15 (<0.001*) | |  |
| PCV | 0.78 (0.989) | |  | |  |
| PED type |  | |  | |  |
| Fibrovascular only | Reference | | Reference | |  |
| Mixed predominantly fibrovascular | 4.08 (0.403) | |  | |  |
| Mixed predominantly serous | 7.25 (0.306) | |  | |  |
| Baseline CRT | −0.34 (0.063) | |  | |  |
| Baseline maximal PED height | 0.16 (0.258) | |  | |  |
| Hyperreflective foci | −49.91 (0.292) | |  | |  |
| **Predictive factors associated with SRF volume change** | | | | | |
| Age | | 4.58 (0.058) | |  | |
| Sex | | 35.11 (0.336) | |  | |
| Duration of aflibercept therapy | | 0.71 (0.488) | |  | |
| MNV type | |  | |  | |
| Type 1 | | Reference | | Reference | |
| Type 3 | | 36.87 (0.516) | |  | |
| PCV | | −47.60 (0.297) | |  | |
| PED type | |  | |  | |
| Fibrovascular | | Reference | | Reference | |
| Mixed predominantly fibrovascular | | −48.89 (0.251) | |  | |
| Mixed predominantly serous | | −153.86 (0.015*) | | −71.22 (0.343) | |
| Baseline CRT | | −0.32 (0.018*) | | −0.19 (0.454) | |
| Baseline maximal PED height | | −0.20 (0.056) | |  | |
| Hyperreflective foci | | −77.67 (0.026*) | | −112.58 (0.010*) | |
| **Predictive factors associated with PED volume change** | | | | | |
| Age | | −0.3 (0.957) | |  | |
| Sex | | 78.56 (0.342) | |  | |
| Duration of aflibercept therapy | | 2.03 (0.384) | |  | |
| MNV type | |  | |  | |
| Type 1 | | Reference | | Reference | |
| Type 3 | | 76.7 (0.521) | |  | |
| PCV | | −295.55 (0.003*) | | −225.72 (0.021*) | |
| PED type | |  | |  | |
| Fibrovascular | | Reference | | Reference | |
| Mixed predominantly fibrovascular | | 2.14 (0.979) | |  | |
| Mixed predominantly serous | | −542.43 (<0.001*) | | −434.08 (0.004*) | |
| Baseline CRT | | −1.03 (0.001*) | | −0.11 (0.784) | |
| Baseline maximal PED height | | −0.84 (<0.001*) | | −0.48 (0.125) | |
| Hyperreflective foci | | - 1. (0.905) | |  | |

***Statistically significant. *CRT* central retinal thickness, *IRF* intraretinal fluid, *MNV* macular neovascularization, *PCV* polypoidal choroidal vasculopathy, *PED* pigment epithelium detachment, *SRF* subretinal fluid

**
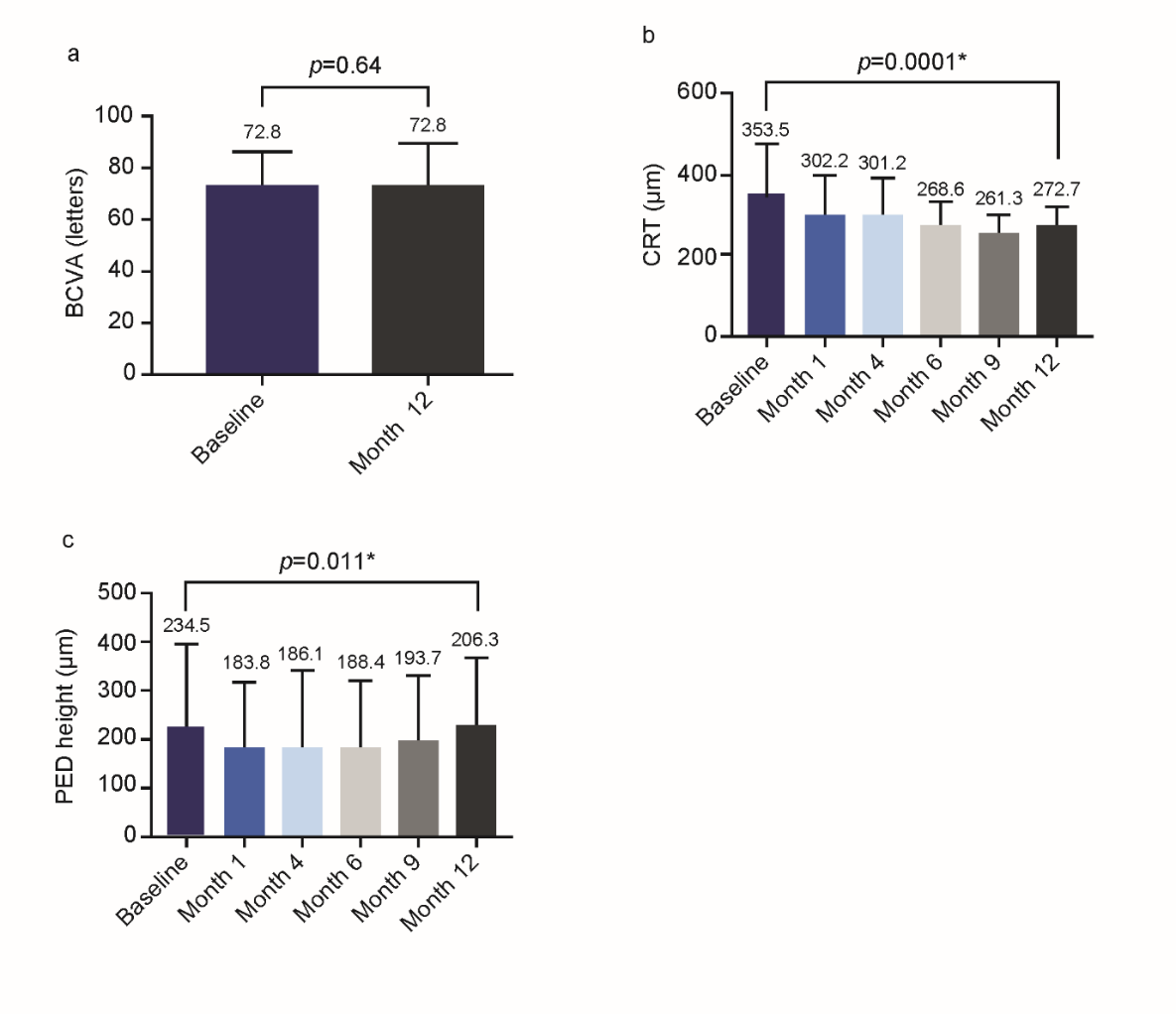
**

**Supplementary Fig. 1** Change in mean ± SD (a) BCVA, (b) CRT, and (c) maximal PED height from baseline to Month 12

***Statistically significant. *BCVA* best-corrected visual acuity, *CRT* central retinal thickness, *PED* pigment epithelium detachment

**
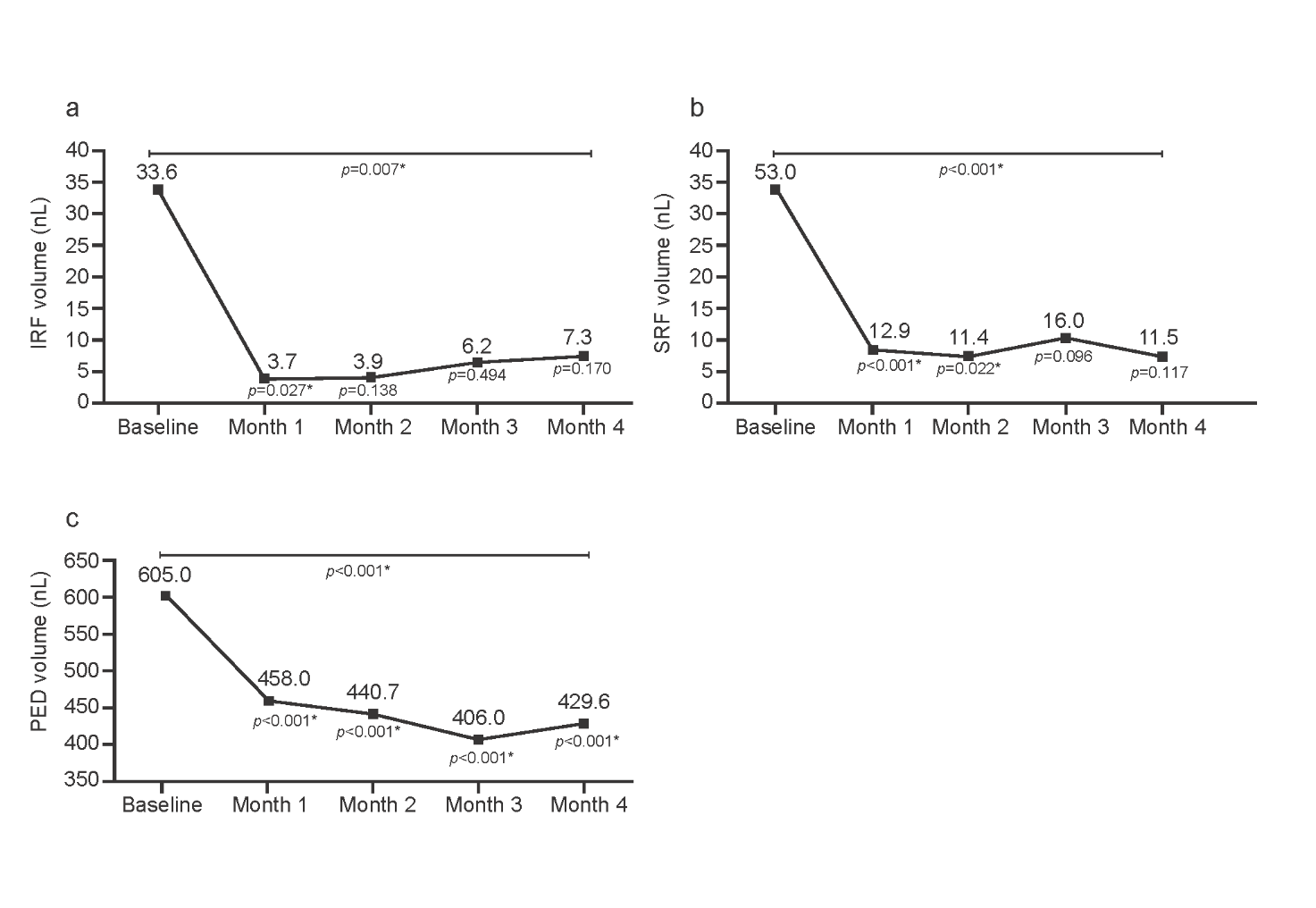
**

**Supplementary Fig. 2** Dynamics of retinal fluid volumes from baseline to Month 4 with faricimab: change in mean (a) IRF, (b) SRF, and (c) PED volumes

***Statistically significant. *IRF* intraretinal fluid, *PED* pigment epithelium detachment, *SRF* subretinal fluid


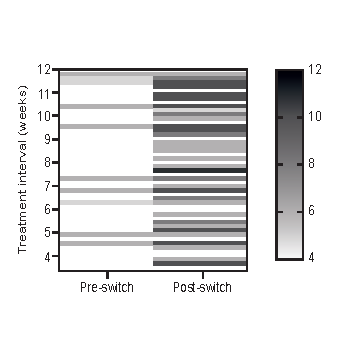


**Supplementary Fig. 3** Maximal fluid-free treatment intervals before (in the 6 months before switching) and after (Month 12) treatment switch
